# Initial diagnosis of herpes zoster ophthalmicus complicated by central retinal artery occlusion and subsequent varicella-zoster virus encephalitis: a case report

**DOI:** 10.3389/fneur.2025.1751103

**Published:** 2026-01-14

**Authors:** Tsai-Wei Lin, Wen-Chien Huang

**Affiliations:** 1 Department of Ophthalmology, Show Chwan Memorial Hospital, Changhua, Taiwan; 2 Department of Neurology, Show Chwan Memorial Hospital, Changhua, Taiwan

**Keywords:** herpes zoster ophthalmicus, retinal artery occlusion, treatment, VZV encephalitis, VZV vasculopathy

## Abstract

**Purpose:**

To report the unusual case of a herpes zoster ophthalmicus (HZO) complicated by central retinal artery occlusion and viral encephalitis.

**Case report:**

A 65-year-old male with HZO who underwent intravenous acyclovir treatment complained of sudden onset blurry vision of his left eye. Central retinal artery occlusion (CRAO) was revealed. We administered methylprednisolone pulse therapy, yet resulted in no visual acuity improvement.

**Conclusion:**

There were only a few cases reported to have CRAO after diagnosed HZO. This cause of viral-related CRAO led to a complicated treatment decision.

## 1 Introduction

Varicella-zoster virus (VZV) establishes latency in sensory ganglia and may reactivate in older or immunocompromised individuals, leading to herpes zoster ophthalmicus (HZO). HZO manifests with vesicular eruptions along the ophthalmic branch of the trigeminal nerve and can cause keratitis, uveitis, or scleritis. Beyond these anterior-segment findings, VZV reactivation occasionally results in severe ischemic and neurological complications through viral-induced vasculopathy (1). Among all forms of zoster, ophthalmic involvement carries the highest risk for subsequent cerebrovascular events (2). VZV vasculopathy arises when the virus spreads transaxonally from ganglionic neurons to arterial walls, infecting the adventitia and media and provoking granulomatous angiitis, intimal hyperplasia, and luminal occlusion (3). In the retina, this same process can present as central or branch retinal artery occlusion (CRAO/BRAO) even without evident necrotizing retinitis (4). Acute retinal ischemia is now recognized as the ocular analogue of acute cerebral stroke and requires emergent evaluation (5, 6). However, in VZV-related ischemia, the mechanism differs from embolic occlusion, involving direct viral endothelial injury and immune-mediated inflammation. Consequently, early antiviral and corticosteroid therapy may be essential to prevent irreversible ocular and central nervous system (CNS) damage. Although several reports have described HZO complicated by central retinal artery occlusion (CRAO) (7–10), most of these cases were limited to ocular vasculopathy without evidence of CNS involvement. Hall et al. (7) first reported CRAO following HZO in an adult patient, and Ahn et al. (8) subsequently described a pediatric case with a similar presentation, while later reports by Camuglia et al. (9) and Ahmad et al. (10) further confirmed that these ischemic complications generally remain confined to the ocular circulation. In contrast, our patient simultaneously developed HZO, CRAO, and cerebrospinal fluid (CSF)–proven varicella-zoster virus encephalitis within the same clinical episode. This constellation underscores the potential for VZV vasculopathy to affect both retinal and cerebral vessels concurrently, rather than sequentially, highlighting the importance of prompt recognition, interdisciplinary collaboration, and timely management.

## 2 Case presentation

A 65-year-old male with a medical history of well-controlled hypertension and type 2 diabetes mellitus presented with a 6-day history of fever, pain, and vesicular eruptions involving the left ophthalmic (V1) division of the trigeminal nerve. On admission, he was alert and had no focal neurological deficits. Baseline laboratory testing on admission demonstrated normal renal function; therefore, intravenous acyclovir was initiated at the standard dosing regimen (750 mg every 8 h).

**Figure 1:**
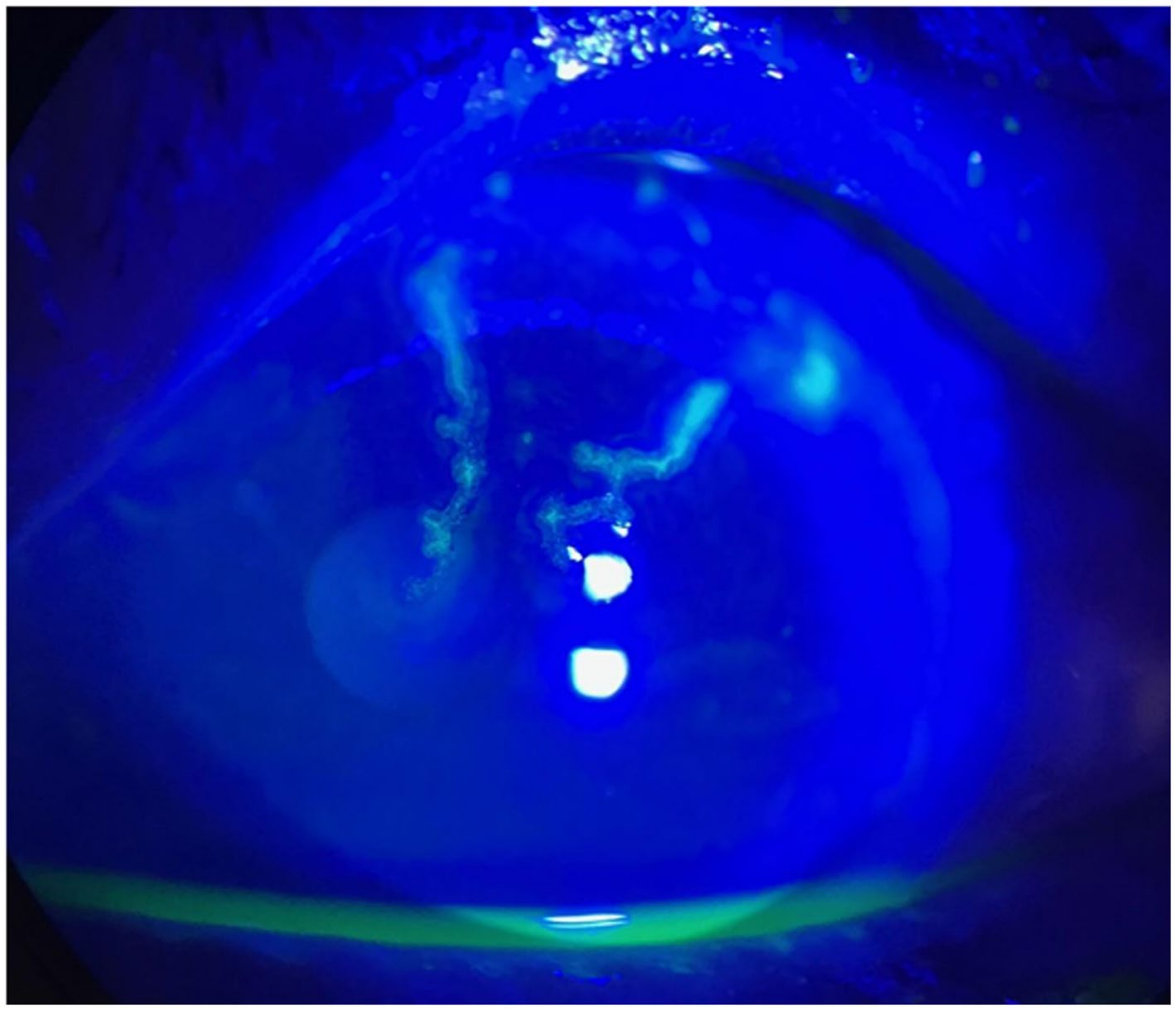
Corneal fluorescein staining revealed pseudo-dendritic corneal lesion indicating VZV keratitis in the left eye.

**Figure 2:**
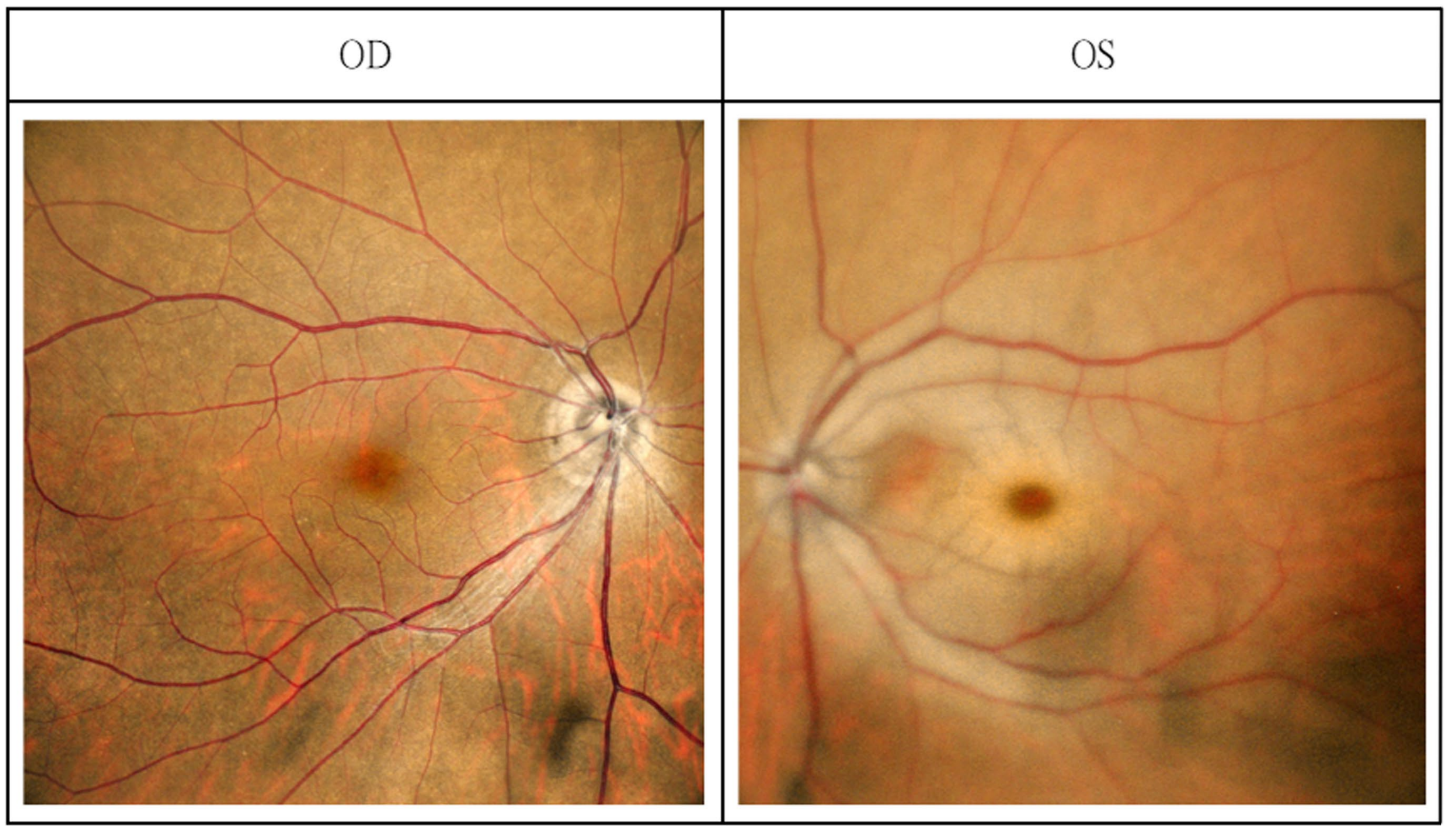
Fundus photography revealed a cherry-red spot in the left eye; the fundus photography of the right eye was normal.

**Figure 3:**
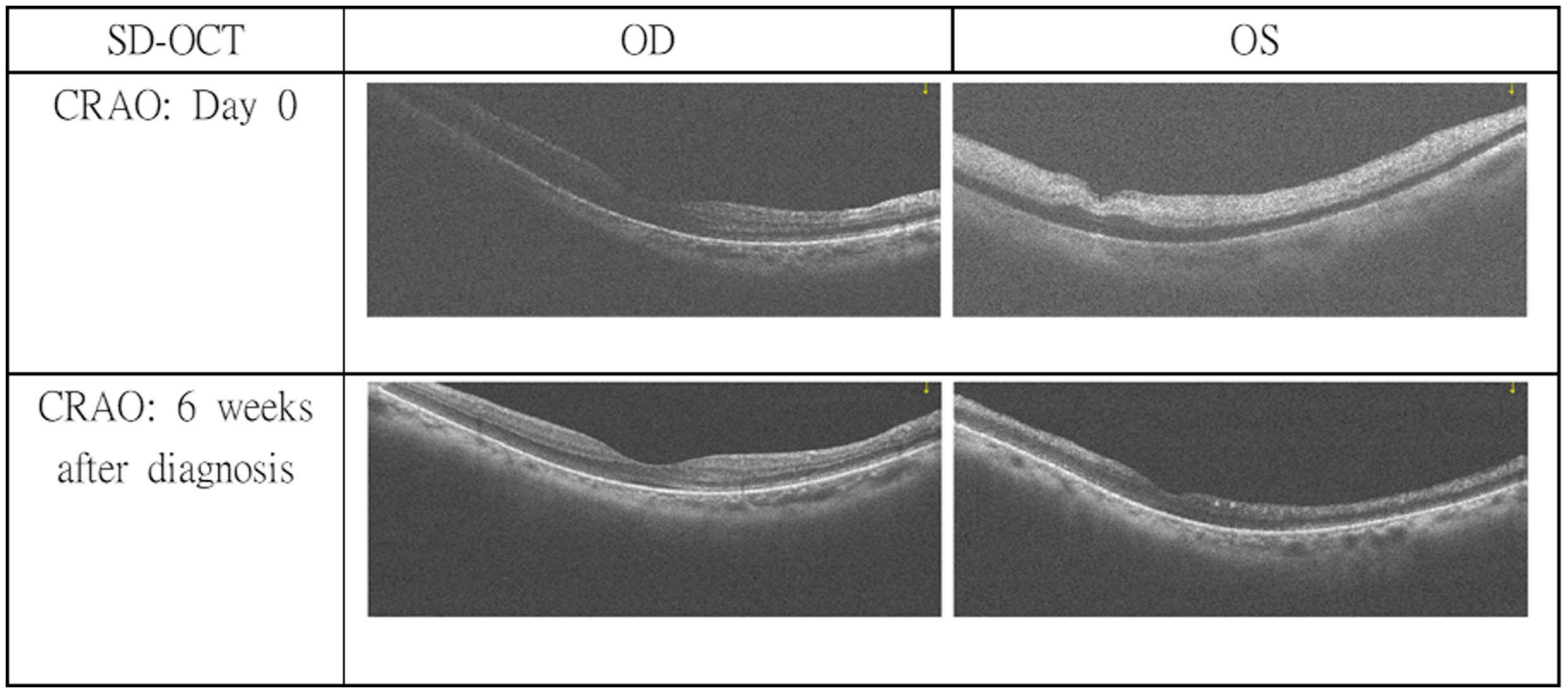
SD-OCT revealed hyper-reflective inner retinal layers indicating retinal edema in the left eye. Thinning of inner retinal layer at 6 week follow-up OCT examination showed a chronic stage of CRAO in the left eye.

Two days after initiation of treatment, the patient awoke at approximately 08:00 with sudden visual loss in the left eye. The last known normal visual status was at approximately 21:00 the previous evening. An urgent ophthalmology consultation was requested due to sudden-onset blurry vision in the left eye. Ocular examination revealed best-corrected visual acuity was measured using a Snellen chart and was 20/20 in the right eye and 20/250 in the left eye. Intraocular pressure was 11 mmHg in the right eye and 13 mmHg in the left. Vesicles and crusts appeared on the left upper eyelid. Corneal fluorescein staining revealed pseudodendritic lesions (Figure 1), and anterior chamber examination showed grade 2 cells, indicating herpes zoster uveitis. Funduscopic examination demonstrated a cherry-red spot at the macula, suggestive of CRAO (Figure 2). The posterior segment showed no vitreous cells or haze. Macular spectral-domain optical coherence tomography (SD-OCT) revealed hyperreflective inner retinal layers consistent with retinal edema, confirming the diagnosis of CRAO (Figure 3).

**Figure 4:**
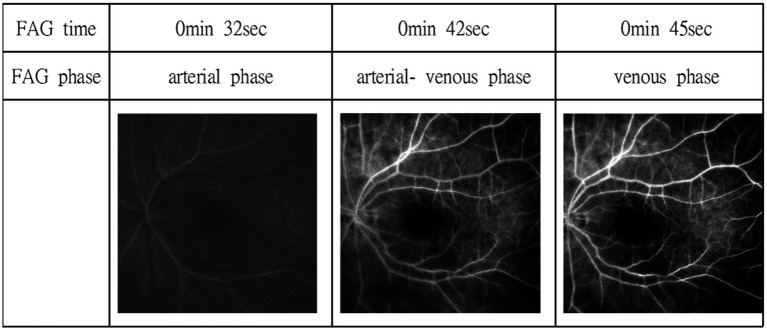
Fluorescein angiography revealed a choroidal and arterial filling delay (28 s), a delayed arterial venous phase (42 s), and a delayed venous phase (45 s). However, no obvious emboli in retinal arteries or filling defect was shown.

Immediate management included ocular massage, hyperventilation, and topical intraocular pressure-lowering agents (dorzolamide) along with prednisolone acetate 1% for the uveitis. Due to the elapsed time window and concerns regarding vasculopathy related to VZV infection, tissue plasminogen activator (tPA) was not administered. Instead, high-dose intravenous methylprednisolone (1,000 mg daily for 5 days) was initiated for presumed VZV-induced CRAO, with limited response. Fluorescein angiography performed a few days later revealed a delayed arterial filling time and arteriovenous transit time, consistent with the CRAO diagnosis (Figure 4). Visual evoked potential (VEP) testing performed after the acute phase revealed delayed P100 latency with reduced amplitude in the left eye, while responses from the right eye were within normal limits, indicating impaired visual conduction along the left visual pathway. A comprehensive stroke workup was performed after the CRAO event, including laboratory evaluation, transthoracic echocardiography, 24-h Holter monitoring, and carotid ultrasonography, all of which revealed no evidence of cardioembolic sources or significant carotid artery disease.

To further clarify the underlying condition, gadolinium-enhanced brain MRI was performed and demonstrated no evidence of acute or subclinical cerebral ischemia, CNS infection, optic neuritis, or radiographic vasculitis. Magnetic resonance angiography (MRA) showed only mild atherosclerotic changes in the basilar artery. Lumbar puncture was performed to evaluate for potential subclinical CNS involvement related to VZV infection, which may occur even in the absence of overt encephalitic signs. Continued acyclovir therapy led to acute kidney injury, manifested by severe metabolic acidosis, altered mental status, and anuria. Emergency hemodialysis and aggressive hydration were initiated, resulting in renal recovery after 5 days. Antiviral therapy was then switched to ganciclovir (250 mg daily) to complete the course. Follow-up CSF analysis was performed to monitor the response to ganciclovir therapy and to guide decisions regarding treatment duration. After 14 days of antiviral therapy, CSF abnormalities persisted, and the treatment course was therefore extended to a total of 21 days based on the overall clinical course and CSF findings.

At follow-up in the ophthalmology outpatient clinic, the patient’s left eye visual acuity remained severely impaired (counting fingers at 10 cm), although the vesicular lesions, corneal findings, and anterior uveitis had resolved. SD-OCT at 6 weeks demonstrated thinning of the inner retinal layers (Figure 3), indicating the chronic stage of CRAO.

## 3 Discussion

CRAO following HZO represents a rare but vision-threatening manifestation of VZV vasculopathy. The underlying pathophysiology is now understood as a viral-induced granulomatous angiitis involving both small and large arteries. After reactivation in the trigeminal ganglion, VZV travels transaxonally along the ophthalmic branch to reach the adventitia of cerebral and retinal arteries, where it infects vascular smooth-muscle and endothelial cells, causing disruption of the internal elastic lamina, intimal proliferation, and luminal thrombosis (3, 10). Histopathologic and molecular analyses have identified VZV antigens and DNA within affected vessels, confirming direct viral infection rather than embolic occlusion (11).

Several reports have described HZO complicated by CRAO, typically as an isolated ocular vasculopathy without CNS involvement (7–10). Hall et al. (7) provided the earliest adult case linking HZO with CRAO, followed by Ahn et al. (8), who reported a pediatric patient demonstrating similar retinal arterial occlusion. Camuglia et al. (9) and Ahmad et al. (10) later described CRAO following HZO. In these reports, CSF evaluation for VZV was not performed or not reported; therefore, concomitant CNS involvement could not be definitively excluded. Collectively, the existing literature suggests that ocular VZV vasculopathy has most often been described as clinically localized to the retina and choroid, although systematic assessment for CNS involvement was lacking.

In contrast, our patient manifested simultaneous HZO, CRAO, and cerebrospinal-fluid–proven VZV encephalitis during a single clinical episode, indicating a more widespread “pan-vasculopathy.” Importantly, ancillary angiographic findings further supported a vasculopathic rather than embolic mechanism of retinal ischemia. Fluorescein angiography demonstrated delayed choroidal filling without visible retinal emboli, an angiographic pattern more consistent with an arteritic cause of CRAO and supportive of an underlying inflammatory vasculopathy (12).

While previous series have documented delayed cerebral infarction or vasculitis weeks to months after HZO (13), concurrent ocular and encephalitic involvement is exceptionally uncommon. Bandeira et al. (14) described that HZO can be associated with both ocular and cerebral vasculopathy, supporting the concept that VZV may affect multiple vascular territories concurrently. Our case extends this spectrum by providing clinico-virologic evidence of concurrent ocular and encephalitic disease, emphasizing that these manifestations likely represent points along a unified pathogenic continuum rather than sequential complications.

Management of VZV-related vasculopathy is guided by observational data and expert consensus. High-dose intravenous acyclovir (10–15 mg/kg every 8 h for minimum 14 days) remains the therapeutic cornerstone and should be initiated promptly upon clinical suspicion for patient having vasculopathy (6, 11). Corticosteroids are frequently administered concurrently with intravenous acyclovir to suppress vascular inflammation and intimal hyperplasia (13). Based on their clinical experience, Gilden and colleagues recommended a short course of oral prednisone (1 mg per kilogram of body weight daily for 5 days) given in conjunction with intravenous acyclovir to further reduce arterial wall inflammation (11). This regimen, reiterated in subsequent reviews by Nagel and Gilden, supports the use of combined antiviral and corticosteroid therapy to achieve anti-inflammatory control (15). Although controlled data are lacking, pulse-dose corticosteroid therapy has been described in cases of severe vasculitic inflammation or CNS involvement, with methylprednisolone administered at a dose of 1,000 mg daily for 3 to 5 days (9, 10). In such reports, vascular inflammation often stabilized or regressed after treatment; however, improvement in visual acuity was limited. In our patient, early antiviral therapy stabilized neurologic function but failed to restore vision, consistent with previous observations that CRAO results in irreversible inner-retinal necrosis once arterial blockage is complete.

Although tPA is a recognized treatment for non-arteritic CRAO within a 4.5-h window (16, 17), its role in VZV–related CRAO remains uncertain. In one reported case involving an immunocompromised patient with HZO, tPA was administered within the therapeutic window, and visual acuity improved from 20/400 to 20/100 (18). Given the multifactorial pathophysiology of VZV vasculopathy, which includes both inflammatory and prothrombotic mechanisms, there is currently no consensus regarding the safety or efficacy of thrombolytic therapy in this setting.

In summary, our patient’s presentation illustrates a rare but pathophysiologically coherent syndrome of retinal VZV vasculopathy accompanied by CSF–proven CNS VZV infection. Early recognition and aggressive antiviral therapy, accompanied by corticosteroids—ranging from short oral courses to high-dose pulse regimens in extensive disease—are essential to prevent irreversible neurological and visual damage. This case broadens the recognized clinical spectrum of VZV infection, demonstrating that what may initially appear as isolated ocular disease can in fact represent a manifestation of systemic neurovasculitis.

## Data Availability

The original contributions presented in the study are included in the article/supplementary material, further inquiries can be directed to the corresponding author.

## References

[1] Liesegang TJ. Herpes zoster ophthalmicus natural history, risk factors, clinical presentation, and morbidity. Ophthalmology. (2008) 115:S3–S12. doi: 10.1016/j.ophtha.2007.10.009, 18243930

[2] Lu P Cui L Zhang X. Stroke risk after varicella-zoster virus infection: a systematic review and meta-analysis. J Neurovirol. (2023) 29:449–59. doi: 10.1007/s13365-023-01144-0, 37219811

[3] Nagel MA. Varicella zoster virus vasculopathy: clinical features and pathogenesis. J Neurovirol. (2014) 20:157–63. doi: 10.1007/s13365-013-0183-9, 23918503 PMC3872206

[4] Sutra P Pokawattana I. Retinal vasculopathy following varicella zoster virus infection. Curr Opin Ophthalmol. (2022) 33:557–63. doi: 10.1097/ICU.0000000000000899, 36165416

[5] Biousse V Newman NJ. Ischemic optic neuropathies. N Engl J Med. (2015) 372:2428–36. doi: 10.1056/NEJMra1413352 26083207

[6] Biousse V Nahab F Newman NJ. Management of acute retinal ischemia: follow the guidelines! Ophthalmology. (2018) 125:1597–607. doi: 10.1016/j.ophtha.2018.03.054, 29716787

[7] Hall S Carlin L Roach ES McLean WT Jr. Herpes zoster and central retinal artery occlusion. Ann Neurol. (1983) 13:217–8. doi: 10.1002/ana.410130226, 6830188

[8] Ahn M Cho NC. Central retinal artery occlusion in herpes zoster ophthalmicus. J Pediatr Ophthalmol Strabismus. (2002) 39:123–4. doi: 10.3928/0191-3913-20020301-16, 11911544

[9] Camuglia JE Beltz JE Khurana K Hall AJ. An unusual cause of visual loss after herpes zoster ophthalmicus: a case report. Cases J. (2010) 3:17. Epub 2010/02/26. doi: 10.1186/1757-1626-3-17, 20180950 PMC2829517

[10] Ahmad SS Suan ALL Alexander SM. Herpes zoster ophthalmicus, central retinal artery occlusion, and neovascular glaucoma in an immunocompetent individual. J Ophthalmic Vis Res. (2019) 14:97–100. doi: 10.4103/jovr.jovr_65_17, 30820294 PMC6388535

[11] Gilden D Cohrs RJ Mahalingam R Nagel MA. Varicella zoster virus vasculopathies: diverse clinical manifestations, laboratory features, pathogenesis, and treatment. Lancet Neurol. (2009) 8:731–40. doi: 10.1016/S1474-4422(09)70134-6, 19608099 PMC2814602

[12] Dagra A Lucke-Wold B McGrath K Mehkri I Mehkri Y Davidson CG . Central retinal artery occlusion: a review of pathophysiological features and management. Stroke. (2024) 4:e000977. doi: 10.1161/SVIN.123.000977 PMC12778453 41586062

[13] Nagel MA Gilden D. Update on varicella zoster virus vasculopathy. Curr Infect Dis Rep. (2014) 16:407. doi: 10.1007/s11908-014-0407-z, 24819870 PMC4112991

[14] Bandeira F Roizenblatt M Levi GC Freitas D Belfort R Jr. Herpes zoster ophthalmicus and varicella zoster virus vasculopathy. Arq Bras Oftalmol. (2016) 79:126–9. doi: 10.5935/0004-2749.20160038, 27224081

[15] Nagel MA Gilden D. Neurological complications of varicella zoster virus reactivation. Curr Opin Neurol. (2014) 27:356–60. doi: 10.1097/WCO.0000000000000092, 24792344 PMC4189810

[16] Nedelmann M Graef M Weinand F Wassill KH Kaps M Lorenz B . Retrobulbar spot sign predicts thrombolytic treatment effects and etiology in central retinal artery occlusion. Stroke. (2015) 46:2322–4. doi: 10.1161/STROKEAHA.115.009839, 26111890

[17] Mac Grory B Schrag M Biousse V Furie KL Gerhard-Herman M Lavin PJ . Management of central retinal artery occlusion: a scientific statement from the American Heart Association. Stroke. (2021) 52:e282–94. doi: 10.1161/STR.0000000000000366, 33677974

[18] Gunturu M Gosi SK Kanduri S Garla V. Varicella zoster meningitis, optic neuritis preceding the development of posterior outer retinal necrosis, and central retinal artery occlusion in a HIV patient. Case Rep Med. (2019) 2019:4213162. doi: 10.1155/2019/4213162, 31467556 PMC6701357

